# A Comparison of parenting stress between mothers of children with spina bifida and able bodied controls

**DOI:** 10.1186/1743-8454-7-S1-S28

**Published:** 2010-12-15

**Authors:** Lai Choo Ong, Nazli AR Norshireen, Vijayalakshmi Chandran

**Affiliations:** 1 Department of Pediatrics, Universiti Kebangsaan Malaysia Medical Centre, Jalan Yaacob Latif, 56000 Kuala Lumpur, Malaysia; 2 Department of Pediatrics, Institute of Pediatrics, Jalan Raja Muda, 50300 Kuala Lumpur, Malaysia

## Background

To compare parenting stress between mothers of children with spina bifida and mothers of able-bodied controls, and to explore factors other than the disease that moderate stress.

## Materials and methods

81 mothers of children with spina bifida and 69 mothers of children with acute, non-disabling illnesses aged 1-18 years completed the Parenting Stress Index Short Form (PSI/SF) and General Health Questionnaire-12 (GHQ). Each child’s adaptive skills was assessed using the Vineland Adaptive Behaviour Scales (VABS). Medical and sociodemographic data were collected from a combination of case notes’ reviews and direct interviews. Multiple regression analysis was used to determine factors related to Parental Distress (PD), Parent-Child Dysfunctional Interaction (P-CDI) and Difficult Child (DC) sub domains of the PSI.

## Results

Compared to controls, mothers of children with spina bifida had lower educational levels and were more likely to be the main caregiver and not working. They also had significantly higher mean scores for the GHQ, Total PSI/SF and the PD (Parent Domain), DC (Difficult Child) and P-CDI (Parent-Child Dysfunctional Interaction) sub scales. Children with spina bifida had lower VABS scores, indicating poorer adaptive skills, than controls. Single parent status, having a child with spina bifida and higher Life Stress scores were associated with higher PD scores. Single parent status, higher Life stress and GHQ scores were associated with higher DC scores. The only factor associated with higher P-CDI scores was lower VABS scores.

## Conclusions

Factors such as life stress events, single parent status, maternal mental health status and the child’s adaptive skills appear to moderate the impact of spina bifida on various aspects of parenting stress.

